# Non-invasive Markers of Liver Fibrosis: Adjuncts or Alternatives to Liver Biopsy?

**DOI:** 10.3389/fphar.2016.00159

**Published:** 2016-06-20

**Authors:** Jun L. Chin, Michael Pavlides, Ahmad Moolla, John D. Ryan

**Affiliations:** ^1^School of Medicine and Medical Science, University College DublinDublin, Ireland; ^2^Oxford Centre for Clinical Magnetic Resonance Research, University of OxfordOxford, UK; ^3^Radcliffe Department of Medicine, University of OxfordOxford, UK; ^4^Translational Gastroenterology Unit, University of OxfordOxford, UK

**Keywords:** hepatology, fibrosis, biomarkers, elastography, MRI

## Abstract

Liver fibrosis reflects sustained liver injury often from multiple, simultaneous factors. Whilst the presence of mild fibrosis on biopsy can be a reassuring finding, the identification of advanced fibrosis is critical to the management of patients with chronic liver disease. This necessity has lead to a reliance on liver biopsy which itself is an imperfect test and poorly accepted by patients. The development of robust tools to non-invasively assess liver fibrosis has dramatically enhanced clinical decision making in patients with chronic liver disease, allowing a rapid and informed judgment of disease stage and prognosis. Should a liver biopsy be required, the appropriateness is clearer and the diagnostic yield is greater with the use of these adjuncts. While a number of non-invasive liver fibrosis markers are now used in routine practice, a steady stream of innovative approaches exists. With improvement in the reliability, reproducibility and feasibility of these markers, their potential role in disease management is increasing. Moreover, their adoption into clinical trials as outcome measures reflects their validity and dynamic nature. This review will summarize and appraise the current and novel non-invasive markers of liver fibrosis, both blood and imaging based, and look at their prospective application in everyday clinical care.

## Introduction

As the gateway to the systemic circulation from the gut, the liver performs several critical functions, coordinating substrate metabolism, and detoxification as well as responding to immune stimuli. Its unique position and vascular supply also leaves it constantly exposed to potential injury, and liver damage, induced by hepatotoxins such as viral hepatitis, alcohol or excess fat, leads to a cycle inflammation and fibrosis. This process involves the deposition of extracellular matrix (ECM), which if degraded results in repair and regeneration; however should the source of liver injury persist, fibrosis may progress, and ultimately evolve to cirrhosis or end-stage liver disease.

Historically, the role of liver biopsy was both for diagnostic and staging purposes. Given the ability to diagnose the majority of liver conditions with blood testing alone, liver biopsy now primarily serves to quantify fibrosis in order to stage disease. This informs the clinician and patient regarding the urgency of initiating therapy (where possible) and prognosis. Indeed, recent longitudinal studies have demonstrated the presence and severity of fibrosis on biopsy as the single most important predictor of outcome in NAFLD, the commonest cause of liver disease in the Western world, highlighting the need for disease staging for an individual patient (Angulo et al., 2015; Ekstedt et al., 2015).

Liver biopsy represents the “gold standard” for the assessment and quantification of liver fibrosis. However, its invasiveness engenders pain and significant potential complications, leading to poor patient acceptance (Cadranel et al., 2000). Other disadvantages include considerable cost (Pasha et al., 1998), and its inherent shortcomings related to sampling error and sample quality from needle biopsy specimens (Sherlock, 1962; Villeneuve et al., 1996; Bedossa et al., 2003; Merriman et al., 2006) have lead to questions regarding its suitability as the reference standard for liver fibrosis (Standish et al., 2006; Bedossa and Carrat, 2009). These factors, along with an acknowledgement of the need to stage disease without reliance on liver biopsy, have lead to an exponential interest in the identification and use of non-invasive markers of liver fibrosis.

The performance of a non-invasive marker of fibrosis is measured using liver biopsy staging of fibrosis as the reference standard. This is typically reported by sensitivity and sensitivity measurements, as well as an area under the receiver operating curve (AUROC) value, which indicates the ability of the marker (at different cut-offs) to correctly classify a patient into a particular category of fibrosis. Values of < 0.7, 0.7−0.8, 0.8−0.9, and >0.9 indicate poor, fair, good, and excellent accuracy, respectively. Serum non-invasive tests can potentially achieve a perfect AUROC value, having been developed and calibrated using liver biopsy as a reference. The same is not true for imaging-based modalities, who are unable to achieve excellent diagnostic accuracy for fibrosis, an important point to consider when interpreting studies (Tsochatzis et al., 2011a). Another issue relates to “spectrum bias,” where different stages of liver fibrosis may be represented unequally within a candidate study, leading to potentially misleading results, highlighting the importance of independent validation of markers.

Non-invasive markers of fibrosis are being incorporated into the routine clinical care of patients with liver disease, although the field continues to evolve. The importance of these markers comes with the stark realization that standard liver biochemistry is of little value in determining the severity of liver fibrosis in primary care (Armstrong et al., 2012). Instead, simple scores based on blood test results can enable a tier system whereby primary care physicians can determine the need for specialist referral for patients (Sheron et al., 2012). With the availability of accurate non-invasive tests, the ability to screen large cohorts for significant liver disease is now becoming a possibility, allowing the assessment of the true burden of liver disease in the general population (Koehler et al., 2016). Moreover, as novel anti-fibrosis therapies enter clinical trials, robust non-invasive markers are crucial to allow effective trial design and obviate the need for multiple, invasive liver biopsies to assess efficacy.

In this review, the types and application of several blood-based non-invasive markers of liver fibrosis are described, followed by a thorough description of current ultrasound based elastography methods and their utility in staging of liver disease. Finally, magnetic resonance imaging techniques to improve staging of liver fibrosis and cirrhosis are considered.

## Non-invasive serological markers

Whilst there have been many non-invasive biomarkers investigated and proposed, an ideal serological test for liver fibrosis which reduces the need to perform invasive investigations such as liver biopsy is still awaited. This ideal serological test would be quick to perform and analyse as well as inexpensive and reproducible. Furthermore, an ideal test would be able to distinguish between distinct entities in liver pathology such as inflammation and fibrosis, be unaffected by impairment in liver function, as well as being able to predict and track disease progression or regression.

When evaluating potential biomarkers, there is a need to ensure consistency in assessment and evaluation, and the related need for simple and robust classification systems (Veidal et al., 2010). This is important as the various serological biomarkers for liver fibrosis described in the medical literature to date have been appraised and validated in differing liver disease contexts, and so in different cohorts of patients. The majority of studies published have been performed in patients with hepatitis C (HCV), with fewer studies performed in patients with hepatitis B (HBV), non-alcoholic fatty liver disease (NAFLD), and alcoholic liver disease (ALD), with fewer still reported in rarer conditions such as in autoimmune liver diseases and hereditary haemochromatosis. As one may expect, these biomarkers perform differently in these differing liver disease contexts. As such, care must be taken to ensure that use of a specific biomarker test at individual patient level has both diagnostic and clinical validity in the context of the patient's illness (Balistreri, 2014). It is equally important to note that most of the biomarkers described in the literature work best in predicting established or advanced fibrosis when compared to the gold standard of liver biopsy, and that biomarkers generally do not perform well in indeterminate stages of liver disease.

To help aid understanding, non-invasive biomarkers of fibrosis may be broadly divided into two classes. Class I markers are direct serum markers reflecting ECM turnover and/or fibrogenic changes at cellular level in the liver. Class II markers of fibrosis are indirect serum markers based on algorithms and mathematical model of varying complexity and derived from changes that occur in liver function (Gressner et al., 2007; Veidal et al., 2010).

### Class I (direct) biomarkers of liver fibrosis

Class I biomarkers are direct markers of fibrosis and reflect the molecular pathogenesis and turnover of liver ECM. The main source of fibrosis in the liver is derived from hepatic stellate cells (HSCs) and myofibroblasts (Korner et al., 1996; Friedman, 2000; Rockey and Friedman, 2006; Gressner et al., 2007; Henderson et al., 2013). Injury to the liver leads to the production of cytokines and to the activation of HSCs initially causing liver inflammation, and then to potentially irreversible change and scarring within the ECM tissue architecture.

Class I markers may be subcategorized into enzymatic markers, collagen (and related) markers, Glycoproteins and matrix-metalloproteinase markers, and Glycosaminoglycan markers (Table 1; Guechot et al., 1996; Murawaki et al., 1999; Walsh et al., 1999; McHutchison et al., 2000; Gressner et al., 2007). Of these various classes, collagen derived markers and glycosaminoglycans (hyaluronic acid) have proven to be the most widely utilized in the development of new fibrosis biomarkers. Few class I markers are used in routine clinical practice as they largely do not directly correlate with whole tissue or body function and so provide data of limited clinical value. As such, their turnaround time is higher, availability is limited to mainly research and specialist settings and investigational costs are generally increased when compared to class II markers. Another limitation is that class I markers are neither all liver derived nor liver specific and so serum measurements do not always directly relate to liver activity. For example, during infection, where acute systemic inflammation co-exists, these markers may also be affected and their absolute values can reflect both fibrinolytic as well as fibrogenic activity.

**Table 1:** **Examples of Class I (direct) serum non-invasive biomarkers for liver fibrosis**.

	**References**		**References**
Metalloproteinases (MMPs)	Murawaki et al., 1999; Walsh et al., 1999	Transforming growth factor (TGF)-β	Flisiak et al., 2002
Tissue inhibitors of metalloproteinases (TIMPs)	Walsh et al., 1999	Microfibril-associated protein 4 (MFAP4)	Molleken et al., 2009
Hyaluronic acid (HA)	Guechot et al., 1996	N Glycans- profiles	Molleken et al., 2009
Laminin	Korner et al., 1996	Procollagen type III amino-terminal peptide (PIIINP)	Guechot et al., 1996
Chitinase-3-like protein 1 (CHI3L1 or YKL-40)	Berres et al., 2009		
Collagen IV, VI	Shahin et al., 1992; Yabu et al., 1994; George et al., 1999		

### Class II (indirect) biomarkers of liver fibrosis

Class II biomarkers provide an indirect reflection of liver ECM activity and fibrosis through the measurement of liver function or injury. As these tests reflect alterations in hepatic function and often correlate with signs and symptoms of illness, they are well established and used routinely in clinical practice to both diagnose and to monitor liver disease.

The broad range of activities the liver performs includes thermostasis, energy balance, carbohydrate, fat and protein metabolism, haemocoagulation, iron and hemoglobin metabolism, immune balance, production of bile acids and hormones, and clearance of toxins (Pratt and Kaplan, 2000; Limdi and Hyde, 2003; Tan et al., 2012; Neuman et al., 2014). Therefore, the number of class II biomarkers that may be tested is extensive.

In daily clinical practice, serological testing for liver function may be broadly categorized into the following categories:

− **Liver enzymes**, including alanine amino transferase (ALT), aspartate amino transferase (AST), alkaline phosphatase (ALP), and gamma glutamyl transferase (GGT)
− **Markers of synthetic function**, such as prothrombin time (PT/INR), bilirubin, haptoglobin, albumin, Apolipoprotein A1, α2-macroglobulin, ceruloplasmin, transferrin and hepcidin
− **Other indirectly measured markers**, such as platelet count, α1-antitrypsin, ferritin, and certain adipokines (adiponectin and leptin; Ding et al., 2005).

As one may expect, although class II markers are more readily available and generally more cost-effective to employ, as proxy markers for fibrosis, their results are not as reliable or accurate as class I markers (Adams et al., 2005). Serum levels of these markers may be affected by a wide range of factors. It is well recognized that ALT levels, commonly thought to indicate the severity of liver disease, are wholly unreliable as a predictor of fibrosis (McCormick et al., 1996) and often within normal range in individuals with advanced disease (Mofrad et al., 2003). Indeed, one recent paper demonstrated a 56% prevalence of NAFLD in 103 volunteers with type 2 diabetes who had normal plasma aminotransferase levels (Portillo-Sanchez et al., 2015).

### Combination or panel non-invasive biomarkers

Whilst individual biomarkers have value as described, the combination of various biomarkers—with varying degrees of complexity—can greatly improve the sensitivity and specificity of these tests. Simple combination tests broadly employ class II biomarkers to indirectly assess liver fibrosis. The simplest of these is the AST/ALT ratio. Afdhal and Nunes summarized a number of studies that assessed its value, with most of these studies undertaken in patients with HCV reporting sensitivities between 53–78% and specificities between 90 and 100% for detecting liver fibrosis (Afdhal and Nunes, 2004). Another simple test is the AST to platelet ratio index (APRI), which uses the platelet count in addition to AST to predict fibrosis, first described in 2003 by Wai and colleagues in patients with HCV (Wai et al., 2003). The APRI score has also been shown to have some value in patients with HBV and PBC, although not in ALD, probably due to the confounding effects of alcohol on platelet suppression (Lieber et al., 2006; Loaeza-del-Castillo et al., 2008; Umemura et al., 2015; Xiao et al., 2015). Other simple indirect combination markers include the PGA (prothrombin time, γ-glutamyl transferase and apolipoprotein A1) and PGAA (PGA plus α-2 macroglobulin) indices, Pohl score and Forn's Index (Imbert-Bismut et al., 2001; Pohl et al., 2001; Forns et al., 2002). Another simple combination panel is the NAFLD fibrosis score (NFS), an easily accessible online score which can be used by general practitioners to triage patients with suspected NAFLD in order to determine the need for referral for specialist assessment (Angulo et al., 2007). The AUROC values, sensitivities, specificities, and predictive values of these tests where available for liver fibrosis are provided in Table 2.

**Table 2:** **Combination scores of non-invasive serum biomarkers of liver fibrosis**.

**Index**	**Parameters**	**Year**	**Disease**	***n***	**AUROC for advanced fibrosis**	**Sens**.	**Spec**.	**NPV**	**PPV**	**Lead author**
AST/ALT Ratio (De Ritis /Sheth)	AST/ALT	1998	HCV	139	−	53^+^	100^+^	81^+^	100^+^	Sheth et al., 1998
		2010	NAFLD	145	0.83	74	78	93	44	McPherson et al., 2010
		2015	PBC	137	0.59	−	−	−	−	Umemura et al., 2015
		2000	HCV	151	−	47^+^	96^+^	88^+^	74^+^	Park et al., 2000
BARD	BMI, AST, ALT, DM	2008	NAFLD	827	0.81	−	−	−	−	Harrison et al., 2008
		2010	NAFLD	145	0.77	89	44	95	27	McPherson et al., 2010
		2011	NAFLD	138	0.67	51	77	81	45	Ruffillo et al., 2011
		2016	NAFLD^∧^	1038	0.76	74	66	−	−	Sun et al., 2016^∧^
Bonacini-index	ALT/AST-ratio, INR, platelet count	1997	HCV	79	−	46	98	−	−	Bonacini et al., 1997
FIB-4	Platelet count, AST, ALT, age	2006	HCV/HIV	830	0.74−0.77	67	71	89	38	Sterling et al., 2006
		2010	NAFLD	145	0.86	85	65	95	36	McPherson et al., 2010
		2009	NAFLD	541	0.80	52	90	−	−	Shah et al., 2009
		2016	NAFLD^∧^	1038	0.85	84	69	−	−	Sun et al., 2016^∧^
		2015	PBC	137	0.71	−	−	−	−	Umemura et al., 2015
Fibrometer test	Platelet count, prothrombin index, AST, α2-macro-globulin, hyaluronic acid, urea, age	2005	HCV/HBV	383	0.89^**^	81^**^	84^**^	77^**^	86^**^	Cales et al., 2005
Fibrometer A	Prothrombin index,' α2 macroglobulin, hyaluronic acid, age	2005	ALD	95	0.96^**^	92^**^	93^**^	83^**^	97^**^	Cales et al., 2005
		2008	ALD	103	0.88	−	−	−	−	Nguyen-Khac et al., 2008
		2009	ALD	218	0.83	−	−	−	−	Naveau et al., 2009
Fibrotest (FT)	Haptoglobin, α2-macro-globulin, apolipoprotein A1. GGT, bilirubin, age, gender	2001	HCV	339	0.87	75	85	80	80	Imbert-Bismut et al., 2001
		2014	HBV^∧^	2494	0.84^**^	61^**^	80^**^	−	−	Salkic et al., 2014^∧^
		2009	ALD	218	0.83	−	−	−	−	Naveau et al., 2009
		2008	ALD	103	0.8	−	−	−	−	Nguyen-Khac et al., 2008
		2006	NAFLD	267	0.81	92	71	98	33	Ratziu et al., 2006
		2003	HCV	352	0.73	−	−	−	−	Poynard et al., 2003
Forns-index	Age, platelet count, GGT, cholesterol	2002	HCV	476	0.81−0.86^**^	94^**^	51^**^	96^**^	40^**^	Forns et al., 2002
Hepascore	Bilirubin, GGT, Hyaluronic acid, α2-macroglobulin, age, gender	2005	HCV	221	0.9−0.96	74−81	88−95	95−98	−	Adams et al., 2005
		2009	ALD	218	0.83	−	−	−	−	Naveau et al., 2009
		2008	ALD	103	0.83	−	−	−	−	Nguyen-Khac et al., 2008
Hui	Body mass index (BMI), platelet count, serum albumin, and total bilirubin	2005	HBV	235	0.79	88	50	92	38	Hui et al., 2005
Leroy-score	PIIINP, MMP-1	2004	HCV	194	0.88	58	92	−	91	Leroy et al., 2004
NAFLD Fibrosis Score (NFS)	Age, BMI, platelets, albumin, AST/ALT, IFG /diabetes	2007	NAFLD	733	0.82−0.88	77−82	71−77	88−93	52−56	Angulo et al., 2007
		2010	NAFLD	145	0.81	33−78	58−98	86−92	30−79	McPherson et al., 2010
		2016	NAFLD^∧^	1038	0.84	77	70	−	−	Sun et al., 2016^∧^
Fibrospect-II	Hyaluronic acid, TIMP-1, α2-macroglobulin	2004	HCV	696	0.82−0.83^**^	77−83^**^	66−73^**^	−	74^**^	Patel et al., 2004
PGA-index	Prothrombin time, GGT, apolipoprotein A1									
		1993	Mixed	169	−	91^+^	81^+^	−	−	Teare et al., 1993
		2008	ALD	103	0.84	−	−	−	−	Nguyen-Khac et al., 2008
PGAA-index	Prothrombin time, GGT, apolipoprotein A1, α2-macroglobulin	1994	ALD	525	−	79^+^	89^+^	−	−	Naveau et al., 1994
		2008	ALD	103	0.86	−	−	−	−	Nguyen-Khac et al., 2008
Pohl score	AST/ALT-ratio, platelet count	2001	HCV	221	−	41	99	93	85	Pohl et al., 2001
ELF score	Hyaluronic acid, TIMP-1, age, MMP-3	2004	HCV	496	0.77	95	29	95	28	Rosenberg et al., 2004
		2004	NAFLD	61	0.87	89	96	98	80	
		2004	ALD	61	0.94	93	100	86	100	
		2008	NAFLD	192	0.90	80	90	94	71	Guha et al., 2008
		2008	PBC	161	0.75	−	−	−	−	Mayo et al., 2008
		2014	Mixed^∧^	1645	0.87	78	76	−	−	Xie et al., 2014^∧^
SHASTA	HA, AST, albumin	2005	HCV-HIV	95	0.87	50	94	83	76	Kelleher et al., 2005
Fibrosis probability-index, FPI	Age, AST, cholesterol, insulin resistance (HOMA), past alcohol intake	2004	HCV	302	0.77−0.84^**^	96^**^	44^**^	93^**^	61^**^	Sud et al., 2004
APRI score	AST, platelet count	2003	HCV	270	0.8−0.88^**^	41−91^**^	47−95^**^	61−88^**^	64−86^**^	Wai et al., 2003
		2010	NAFLD	145	0.67	27	89	84	37	McPherson et al., 2010
		2008	ALD	103	0.43	−	−	−	−	Nguyen-Khac et al., 2008
		2015	PBC	137	0.84	−	−	−	−	Umemura et al., 2015
		2008	HBV	264	0.86^**^	87^**^	66^**^	81^**^	74^**^	Shin et al., 2008
Zeng	alpha2-macroglobulin, age, gamma glutamyl transpeptidase, and hyaluronic acid	2005	HBV	372	0.77−0.84^**^	35−95^**^	44−95^**^	51−86^**^	70−91^**^	Zeng et al., 2005

*ALT, alanine aminotransferase; AST, aspartate aminotransferase; INR, international normalized ratio; GGT, γ-glutamyltransferase; PIIINP, N-terminal propeptide of type III procollagen; TIMP, tissue inhibitors of metalloproteinases; MMP, matrix metalloproteinases*.

** *Values are for prediction of significant fibrosis*.

+ *Values are for prediction of cirrhosis*.

∧ *Metaanalysis*.

More complex combination biomarkers involve a panel of tests or complex algorithms, some of which are patented in the assessment liver fibrosis. These include the Fibrotest/Fibrosure®, Fibrometer®, and Hepascore® (Imbert-Bismut et al., 2001; Adams et al., 2005; Cales et al., 2005). As these tests generally include some class I markers as well as class II markers (Table 2), they have enhanced diagnostic accuracy, however this comes at an increased cost and reduced test availability in some cases, while also demanding greater physician time and effort to calculate and subsequently to interpret. Nevertheless, as these panels continue to improve, they are gaining increased acceptance in clinical practice, and large longitudinal studies have demonstrated their ability to predict prognosis and liver-related outcomes. For example, the Fibrometer® and FIB-4 showed good ability to predict significant liver-related events [AUROC values of 0.88 (0.83–0.91) and 0.87 (0.81–0.92)] in 373 patients with HCV over a median 9.5 year follow up, better than that predicted by fibrosis grade (Boursier et al., 2014). The Enhanced Liver Fibrosis (ELF) test similarly demonstrated prognostic capabilities in 457 patients with chronic liver disease followed a median over 7 years to predict death and clinical outcomes (Parkes et al., 2010). Moreover, a number of studies have now shown the value of the simpler investigations such as the NFS, the FIB-4 and APRI not only in the assessment of liver fibrosis, but also in estimating prognosis (Vallet-Pichard et al., 2007; Treeprasertsuk et al., 2013; Vergniol et al., 2014; Xiao et al., 2015). These findings are important considerations for the application of non-invasive markers with well-validated diagnostic and prognostic ability as outcome measures in clinical trials, obviating the need for repeated liver biopsies thereby enhancing recruitment and study efficiency. In routine clinical practice, the use of these scores alone or in combination can also significantly reduce the need for a liver biopsy, increasing the appropriateness and diagnostic yield from the remaining biopsies that are performed. For instance, Sebastiani and co-workers found that the combination of the APRI test with the Fibrotest-Fibrosure in 2035 patients with chronic HCV infection reduced the need for liver biopsy in 50–80% of cases, with high (>90%) diagnostic accuracy for significant fibrosis and cirrhosis (Sebastiani et al., 2009).

In summary, the quest to find an ideal non-invasive serological biomarker or combination of markers to diagnose and predict the outcome of liver fibrosis is ongoing, with considerable progress made over the past decade. Nevertheless, these investigations remain imperfect, particularly for the detection of intermediate fibrosis grades, and require judicious use tailored to the individual case, and detailed understanding to ensure their use is valid. As these investigations continue to be refined and improved, the need for staging liver biopsy will continue to decline over time.

## Non-invasive ultrasound elastography

### Ultrasound based elastography for the assessment of liver fibrosis

Ultrasound based elastography has revolutionized the assessment of liver fibrosis over the last decade. The concept of elastography for the assessment of fibrosis is simple and stems from applying an external force to soft tissue, compressing the tissue, and thereby generating a shear wave which can be measured (Ophir et al., 1991). The speed of the shear wave propagating through the tissue corresponds to the tissue elasticity based on Young's modulus and hence, the amount of tissue fibrosis (Sandrin et al., 2003).

To date, a number of different types of ultrasound based elastography methods have been developed to assess liver fibrosis and diagnose cirrhosis (Thiele et al., 2015). One can broadly distinguish these elastography methods into two main groups: one-dimensional ultrasound elastography—transient elastography (TE); or two-dimensional (or B-mode) ultrasound which utilizes conventional ultrasound imaging - acoustic radiation force impulse (ARFI) imaging or point shear wave elastography (pSWE), real-time elastography (RTE), and real-time 2D shear wave elastography (2D-SWE). It is likely that some of these ultrasound-based elastography instruments will improve in time with the advent of novel technology.

Elastography measures tissue stiffness. Assessing liver fibrosis with ultrasound-based elastography only measures a small component of tissue stiffness. Many other factors can affect liver tissue stiffness and have been shown to affect the accuracy of assessing liver fibrosis with elastography. These factors were initially studied in TE and include: Inflammation from acute hepatitis (Arena et al., 2008a; Oliveri et al., 2008; Sagir et al., 2008); cholestasis from biliary tract obstruction (Millonig et al., 2008); blood congestion due to hepatic outflow obstruction and portal hypertension(Millonig et al., 2010; Shi et al., 2013); and food intake (Mederacke et al., 2009; Arena et al., 2013; Berzigotti et al., 2013) Another example that elastography measures more than just tissue fibrosis is the utilization of elastography to measure spleen stiffness in patients with cirrhosis; where spleen stiffness correlates with hepatic venous pressure gradient (HVPG; Colecchia et al., 2012; Sharma et al., 2013; Takuma et al., 2013) and reduces after liver transplantation (Chin et al., 2015). Although not studied extensively in other ultrasound-based elastography methods, all of these factors are thought to apply when interpreting liver stiffness measurements obtained with newer techniques (Bota et al., 2013a; Popescu et al., 2013).

### Transient elastography

Transient elastography (Fibroscan®, Echosens, Paris) is the first ultrasound-based elastography developed and is now a well-established non-invasive method of diagnosing and staging hepatic fibrosis (Sandrin et al., 2003). TE utilizes a mono-dimensional ultrasound to determine liver stiffness by measuring the velocity of low frequency elastic shear waves propagating through the liver. TE can be performed in a short time (typically less than 5 min) and has excellent intra- and inter- observer variability (Fraquelli et al., 2007; Boursier et al., 2008).

The Fibroscan® probe consists of a 3.5 MHz ultrasound transducer installed on the axis of a low amplitude vibrator (frequency of 50 Hz and amplitude of 2 mm peak-to-peak). To obtain liver stiffness measurements, the tip of the ultrasound transducer is placed in the right intercostal area, at the level of the right lobe of liver. When activated, the vibrator generates an elastic shear wave to the liver while the ultrasound transducer performed a series of ultrasound acquisitions (transmission/ reception) with a repeat frequency of 4 kHz. A median value of at least 10 successful measurements with an Interquartile range (IQR) of ≤ 30% from the median and success rate of ≥60%, were considered as reflective of the liver stiffness or shear modulus of the liver. This value is expressed in kilopascals (kPa).

TE has been extensively studied for the assessment of fibrosis in many chronic liver diseases, particularly viral hepatitis. The two index studies on TE correlated liver stiffness with the degree of fibrosis (METAVIR staging) on liver biopsy due to chronic HCV infection (Castéra et al., 2005; Ziol et al., 2005). Since then, a number of comparable studies in HCV validated this finding (Coco et al., 2007; Arena et al., 2008b; Lupsor et al., 2009; Wang et al., 2009; Degos et al., 2010; Zarski et al., 2012; Afdhal et al., 2015), and a meta-analysis had reported excellent diagnostic accuracy for HCV-related cirrhosis with an AUROC of 0.95 (0.87–0.99; Shaheen et al., 2007). Similar studies were performed for patients with chronic HBV (Oliveri et al., 2008; Chan et al., 2009; Kim et al., 2009; Marcellin et al., 2009; Wang et al., 2009; Degos et al., 2010; Sporea et al., 2010; Cardoso et al., 2012; Goyal et al., 2013; Afdhal et al., 2015) and co-infection with HCV/HIV (de Lédinghen et al., 2006; Vergara et al., 2007; Kirk et al., 2009; Sánchez-Conde et al., 2010; Castera et al., 2014), with good correlation between liver stiffness and fibrosis. Furthermore, liver stiffness by TE has been shown to predict liver fibrosis in ALD (Nahon et al., 2008; Nguyen-Khac et al., 2008), NAFLD (Nobili et al., 2008; Yoneda et al., 2008; Lupsor et al., 2010; Wong et al., 2010a; Petta et al., 2011) and chronic cholangitides (notably, PBC and PSC; Corpechot et al., 2006, 2012). The diagnostic performance of TE have been confirmed by several meta-analyses to show excellent diagnostic accuracy for cirrhosis of (>90%), but only good accuracy for fibrosis (>80%; Shaheen et al., 2007; Friedrich-Rust et al., 2008; Stebbing et al., 2010; Tsochatzis et al., 2011b; Chon et al., 2012; Table 3).

**Table 3:** **Diagnostic performance for ultrasound-based elastography methods for the detection of liver fibrosis**.

**Study**	**Aetiology**	**AUROC for *F* > 2 (95%CI)**	**Cutoff for F2**	**Sensitivity (%)**	**Specificity (%)**	**AUROC for F4 (95%CI)**	**Cutoff for F4**	**Sensitivity (%)**	**Specificity (%)**
**TE**			**kPa**				**kPa**		
Talwalkar et al., 2007	All	0.87 (0.83–0.91)	–	70.0 (67.0–73.0)	84.0 (81.0–87.0)	0.96 (0.94–0.97)	–	87.0 (84.0–90.0)	91.0 (89.0–92.0)
Friedrich-Rust et al., 2008	All	0.84 (0.82–0.86)	7.7	–	–	0.94 (0.93–0.95)	13.0	–	–
Stebbing et al., 2010	All	–	7.7	71.9 (71.4–72.4)	82.4 (81.9–82.9)	–	15.1	84.5 (84.2–84.7)	94.7 (94.3–95.0)
Tsochatzis et al., 2011b	All	–	–	79.0 (74.0–82.0)	78.0 (72.0–83.0)	–	–	83.0 (79.0–86.0)	89.0 (87.0–91.0)
Shaheen et al., 2007	HCV	0.83 (0.03–1.00)	8.0	64.0 (50.0–76.0)	87.0 (80.0–91.0)	0.95 (0.87–0.99)	12.5	85.6 (77.8–91.0)	93.2 (90.2–95.3)
Chon et al., 2012	HBV	0.859 (0.857–0.860)	7.9	74.3	78.3	0.929 (0.928–0.929)	11.7	84.6	81.5
**pSWE/ARFI**			**m/s**				**m/s**		
Friedrich-Rust et al., 2012	All	0.87	1.34	79.0	85.0	0.93	1.80	92.0	86.0
Nierhoff et al., 2013	All	0.84 (0.80–0.87)	1.35	–	–	0.91 (0.89–0.94)	1.87	–	–
Bota et al., 2013b	All	0.85 (0.82–0.88)	1.30	74.0 (66.0–80.0)	83.0 (75.0–89.0)	0.93 (0.91–0.95)	1.80	87.0 (79.0–92.0)	87.0 (81.0–91.0)
Guo et al., 2015	All	0.85	–	76.0 (73.0–78.0)	80.0 (77.0–83.0)	0.94	–	88.0 (84.0–91.0)	83.0 (81.0–84.0)
Liu et al., 2015	NAFLD	0.90 (0.84–0.96)	–	80.2 (75.8–84.2)	85.2 (80.8–89.0)	–	–	–	–

*Diagnostic performance for transient elastography (TE) and point shear wave elastography/acoustic radiation force impulse (pSWE/ARFI) for F > 2 and F4 presented in a number of meta-analyses*.

### Point shear wave elastography/acoustic radiation force impulse imaging

pSWE (Elastography point quantification, ElastPQ™, Phillips) and ARFI imaging (Virtual touch tissue quantification™, Siemens) are ultrasound-based elastography techniques which utilize B-mode ultrasound. This elastography technique can be incorporated into conventional ultrasound platforms, such as the iU22xMATRIX by Philips (Amsterdam, Netherlands) and Acuson S2000/3000 by Siemens Healthcare (Erlangen, Germany). Hitachi Aloka (Tokyo, Japan) also recently announced their pSWE method known as Shear Wave Measurement in the Radiological Society of North America Meeting in November 2015. pSWE/ARFI measures the speed of shear waves generated by acoustic pulses that lead to localized displacements of liver tissue (Nightingale et al., 2002). Liver stiffness measurement with pSWE/ARFI is performed at the right lobe of the liver, through the intercostal space, at the same site as TE. After selecting a region of interest, at a depth of 2–8 cm with B-mode ultrasound, the shear-wave velocity is measured within the defined region by using ultrasound tracking beams laterally adjacent to the single push beam (Friedrich-Rust et al., 2009). Similar to TE, pSWE/ARFI have good intra- and inter-observer agreement, with an intra-class correlation coefficient of between 0.84 and 0.87 (Boursier and Cales, 2010; Bota et al., 2012).

pSWE/ARFI has been described in many studies to be comparable to TE (Friedrich-Rust et al., 2009; Lupsor et al., 2009; Yoneda et al., 2010; Ebinuma et al., 2011; Piscaglia et al., 2011; Rifai et al., 2011; Sporea et al., 2012). Like TE, pSWE/ARFI is able to diagnose cirrhosis more accurately than significant fibrosis. Several meta-analyses of pSWE/ARFI confirmed good diagnostic accuracy for significant fibrosis, with mean AUROC of 0.84 to 0.87 and excellent diagnostic accuracy for cirrhosis of 0.91–0.94 (Friedrich-Rust et al., 2012; Bota et al., 2013a; Nierhoff et al., 2013; Guo et al., 2015). Based on data from these meta-analyses, the suggested cut-off values for the diagnosis of significant fibrosis were 1.30–1.35 m/s and for cirrhosis, the values were 1.80–1.87 m/s. A recent meta-analysis of pSWE/ARFI in NAFLD reported good diagnostic accuracy of pSWE/ARFI in assessing significant fibrosis in patients with NAFLD (AUROC of 0.898; Liu et al., 2015; Table 3).

### Real-time elastography

RTE is a qualitative assessment of liver stiffness. For RTE, strain elastography is provided either by the operator applying manual compression (Friedrich-Rust et al., 2008) or compression is applied by the transmitted heartbeat (Koizumi et al., 2011). The reflected ultrasound echoes between the states of compression can be computed to measure displacement within each location of the tissue. Hence, the harder the tissue, the lower the amount of displacement of reflected ultrasound echoes before and under compression. A number of quantitative methods for RTE have been described to improve the comparability of this elastography technique, which include the elastic ratio, elastic index, elasticity score, and Liver Fibrosis Index (Friedrich-Rust et al., 2007; Kanamoto et al., 2009; Tatsumi et al., 2010; Koizumi et al., 2011; Colombo et al., 2012; Ferraioli et al., 2012a; Fujimoto et al., 2013; Yada et al., 2013). The basis of these quantitative methods for RTE is to provide a ratio of stiffness or elasticity. When comparing RTE to other ultrasound elastography methods, a recent meta-analysis reported lower diagnostic accuracy with RTE for significant liver fibrosis and cirrhosis, compared to TE or ARFI (Kobayashi et al., 2015). The marked heterogeneity of RTE studies was reported as one of the main reasons for lower diagnostic performance of RTE.

### Real-time 2D shear wave elastography

Real time 2D-SWE is a relatively new ultrasound elastography method, which combines the initiation of a radiation force in tissue with focused ultrasonic beams and acquisition of transiently propagating resultant shear waves in real-time with a high-frequency ultrasound imaging sequence (Muller et al., 2009). In 2D-SWE, a large color coded elastography map is generated by combining several shear waves over time with rapid ultrasound acquisition (Thiele et al., 2016). 2D-SWE is performed quite similarly to pSWE/ARFI. Both the size and location of the region of interest can be chosen by the operator. Although different to pSWE/ARFI in technology, 2D-SWE is similar to pSWE/ARFI for the clinical user as it is implemented on a commercial B-mode ultrasound platform (Aixplorer®, Supersonic Imaging, Aix en Provence, France).

Liver stiffness measured by 2D-SWE has been shown to correlate with the stages of hepatic fibrosis on liver biopsy in chronic HCV (Ferraioli et al., 2012b), chronic HBV (Leung et al., 2013; Zeng et al., 2014), and alcohol-related liver disease (Thiele et al., 2016). In mixed cohorts of patients with chronic liver disease, several studies have shown similar results (Cassinotto et al., 2014; Jeong et al., 2014; Bota et al., 2015; Deffieux et al., 2015; Gerber et al., 2015; Grgurevic et al., 2015; Samir et al., 2015). There is now increasing evidence that 2D-SWE is a comparable elastography method to TE and pSWE/ARFI (Bavu et al., 2011; Ferraioli et al., 2012a; Leung et al., 2013; Cassinotto et al., 2014; Bota et al., 2015; Gerber et al., 2015; Thiele et al., 2016), although no published meta-analysis confirming these findings exists, as yet.

### Comparative performance of ultrasound based elastography

Based on data from published literature on elastography, the diagnostic performance for significant fibrosis and cirrhosis for TE, pSWE/ARFI and probably 2D-SWE are equivalent. In contrast, RTE has a slightly lower diagnostic accuracy and remains limited to centers with experience with this method (Kobayashi et al., 2015). The diagnostic accuracy (AUROC values) for significant fibrosis using TE, pSWE/ARFI and 2D-SWE ranges between 0.65–0.97, 0.77–0.94, and 0.82–0.99 respectively, with excellent results consistently seen for cirrhosis, ranging between 0.80–0.99, 0.87–0.99, and 0.87–0.99, respectively. One advantage of TE is that it can be performed at the point of care, whereas second-generation elastrography techniques tend to require operators trained in ultrasound, reducing accessibility.

The failure rate of TE has been reported to range from 2.7 to 3.1% in obtaining any measurement, and 11.6 to 15.8% in acquiring unreliable results (Castéra et al., 2010; Wong et al., 2011). For pSWE/ARFI (Bota et al., 2013b) and 2D-SWE (Leung et al., 2013; Poynard et al., 2013; Elkrief et al., 2015), the failure rate for acquiring reliable results has been reported to be significantly lower than TE. Liver stiffness measured with 2D-SWE can be expressed either in kPa at a wide range of values (2–150 kPa) or in the form of shear wave velocity, in m/s. One of the main limitations of ARFI is the narrow range of shear wave velocity measurements of 0.5–4.4 m/s, in contrast to the range of TE of 2.5–75 kPa. This is thought to limit the ability of ARFI to define discriminative cut-off values for certain stages of fibrosis.

Quality criteria for liver stiffness measurements have previously been emphasized for TE to provide consistent and reproducible results. These criteria are less clear for pSWE/ARFI and 2D-SWE. The manufacturer of TE recommends that at least 10 successful liver stiffness measurements are acquired with an IQR of ≤ 30% from the median, and a success rate of ≥60%. The median value is taken as representative of liver stiffness. For pSWE/ARFI, most studies emulate the quality criteria of TE, where 10 valid measurements are performed and the median value deemed as the representative measure. Unreliable results for pSWE/ARFI were defined as an IQR/liver stiffness of greater than 30%. In both TE and pSWE/ARFI, a single shear wave is emitted temporarily at a single frequency for each measure. Hence, maintaining these quality criteria is fundamental to consistent measure of liver stiffness.

For 2D-SWE, these quality criteria do not apply. In 2D-SWE, the ultrasound transducer emits a plurality of pulse waves at increasing depth, using a very wide frequency band ranging from 60 to 600 Hz (Bercoff et al., 2004; Muller et al., 2009). The 2D-SWE transducer synchronously evaluate the velocity of several shear wave fronts over a wide frequency range. A real-time color coded map of elasticity is generated and is superimposed on the standard B-mode image. By selecting a region of interest in the center of the color map, the calculated value is the average of many values within the area (Cassinotto et al., 2014). Therefore, the quality criteria for 2D-SWE are not identical. Although not defined, a number of 2D-SWE studies have suggested the following: standard deviation/median ratio < 0.1–0.3 and depth of measurement < 5.6 cm; stable viscoelasticity map for at least 3 s with a homogenous color in the region of interest of at least 15 mm; and 3 measurements with the mean calculated as representative or using the median when >6 measurements are acquired (Sporea et al., 2013; Yoon et al., 2014; Procopet et al., 2015; Thiele et al., 2016).

Similar to serum biomarkers, ultrasound-based elastography is excellent for the non-invasive diagnosis of cirrhosis but only modestly good for significant fibrosis. When comparing TE with serum biomarkers, a number of studies have shown that both TE and serum biomarkers have equivalent performance for detecting significant fibrosis in HCV (Castéra et al., 2005; Degos et al., 2010; Zarski et al., 2012). Conversely, TE performs better than serum biomarkers for detecting cirrhosis due to HCV, if TE measurement is possible (lower applicability of TE compared to serum biomarkers, 80 vs. 95%; Degos et al., 2010; Zarski et al., 2012). While algorithms combining TE with serum biomarkers improve diagnostic accuracy in assessing fibrosis, particularly in HCV patients (Wong et al., 2010b; Boursier et al., 2011, 2012; Zarski et al., 2012), this strategy do not increase the overall diagnostic accuracy for detecting cirrhosis (Castéra et al., 2009; Boursier et al., 2011; Zarski et al., 2012). Furthermore, the combination of TE with serum fibrosis biomarkers is often advocated to better inform the clinician, allowing an informed judgment regarding the need for liver biopsy, for example where concordance exists in extremes of fibrosis, or discordance in intermediate stages.

Several studies have demonstrated that liver stiffness measured by ultrasound based elastography could predict clinical hepatic decompensation, liver related complication and prognosis (de Lédinghen et al., 2006; Robic et al., 2011; Vergniol et al., 2011; Corpechot et al., 2012; Merchante et al., 2012; Pang et al., 2014). Although a number of reports have described good correlation between liver stiffness and HVPG (Vizzutti et al., 2007; Bureau et al., 2008; Lemoine et al., 2008), the correlation between clinically significant portal hypertension and liver stiffness becomes less significant for HVPG values of >12 mmHg (Vizzutti et al., 2007). One potential explanation is that as cirrhosis progresses, the mechanism for portal hypertension is thought to be less dependent on intrahepatic resistance, and more dependent of the resultant hyperdynamic circulation and splanchnic vasodilatation (Reiberger et al., 2012). At present, ultrasound based elastography cannot replace HVPG for evaluation of portal hypertension or upper gastrointestinal endoscopy for detecting oesophageal varices. However, TE is an invaluable tool to risk stratify patients with clinically significant portal hypertension and identify patients at risk of developing HCC (Latinoamericana, 2015).

## Non-invasive magnetic resonance imaging

### Magnetic resonance techniques for liver fibrosis assessment

Magnetic resonance (MR) imaging techniques are attractive tools for liver fibrosis assessment. In contrast to ultrasound techniques, MR allows deep penetration into tissues, and unlike computed tomography avoids ionizing radiation. Repeated assessments can therefore be performed, without safety concerns. Furthermore, MR can assess the whole liver, eliminating sampling errors and can provide additional information on anatomy. There is now extensive experience in the use of MR for the evaluation of diffuse liver disease due to liver fat or iron deposition. Both magnetic resonance imaging (MRI) and magnetic resonance spectroscopy (MRS) were found to be more accurate than histological assessment by a pathologist or by morphometry. (Roldan-Valadez et al., 2010; Raptis et al., 2012) MR has been used to study the epidemiology of NAFLD (Browning et al., 2004; Szczepaniak et al., 2005; Wong et al., 2012) and more recently it has been used as a surrogate end-point in two NAFLD clinical trials (Le et al., 2012; Loomba et al., 2015). MR techniques are also regarded as the gold standard for quantification of liver iron as they have shown an excellent inverse association with biochemically quantified hepatic iron concentration (Gandon et al., 2004; St Pierre et al., 2005). More recently, MR techniques for the assessment of liver fibrosis have been developed and have produced some promising results.

### Magnetic resonance elastography

Similar to ultrasound based elastography techniques, magnetic resonance elastography (MRE) can determine liver stiffness by analysis of mechanical waves propagating through the liver. The technique requires the positioning of an external passive driver on the patient's body adjacent to the liver. The passive driver is connected to a pneumatic active driver placed outside the scanner room which generates the mechanical waves that are propagated through the liver.

#### MRE for liver fibrosis evaluation

In a large prospective single center study of 96 patients with mixed liver disease aetiologies (63% HCV; 8% NAFLD), MRE had excellent diagnostic accuracy for the differentiation of all stages of fibrosis, with an area under the receiver operating characteristic curve (AUROC) for ≥F3 and F4 of 0.99 and 1.00 respectively. (Huwart et al., 2008) Similar results have been obtained in patients with chronic HBV and HCV, (Ichikawa et al., 2012; Venkatesh et al., 2014a) and MRE was also shown to be closely related to morphometric quantification of liver fibrosis (*r* = 0.78; *p* < 0.001; Venkatesh et al., 2014b). In a meta-analysis of 697 individual patient data from 12 studies, MRE was found to have a good diagnostic performance for all stages of liver disease (with excellent accuracy in advanced disease; AUROC for ≥F3 and F4 of 0.93 and 0.92, respectively; Singh et al., 2015). A separate meta-analysis of 13 studies containing 989 patients (but not individual patient data) found pooled AUROC of 0.96 and 0.98 for the diagnosis of fibrosis stage ≥F3 and F4 respectively (Su et al., 2014). Despite these promising results, some concerns still exist regarding the true diagnostic accuracy of MRE, as some of the studies suffer from spectrum bias due to underrepresentation of patients with intermediate stages of fibrosis. For example, the distribution of fibrosis stages in one study was: F0 (*n* = 28), F1 (*n* = 12), F2 (*n* = 6), F3 (*n* = 6), F4 (*n* = 20; Wang et al., 2011).

#### MRE for the evaluation of NALFD

MRE was found to be useful in the assessment of fibrosis in patients with NAFLD, with AUROC 0.92 and 0.89 for the diagnosis of fibrosis stage ≥F3 and F4 respectively (Loomba et al., 2014). Mixed results have been reported for use of MRE in the diagnosis of non-alcoholic steatohepatitis (NASH). Animal and retrospective human studies (Salameh et al., 2009; Chen et al., 2011), have shown some promise, although in a prospective study, MRE only had a modest diagnostic accuracy for NASH (AUROC of 0.73; Loomba et al., 2014).

#### Predictive value of MRE in patients with cirrhosis

A recent study examined the value of MRE in predicting clinical outcomes in 430 patients with cirrhosis, with follow up data on 167 patients whose cirrhosis decompensated during the study. The authors showed that liver stiffness (LS) measured by MRE was independently associated with the presence of decompensation at baseline. Furthermore, a liver stiffness ≥5.8 kPa was a significant risk for decompensation (hazard ratio 4.96; 95%CI 1.4-17.0) in patients who had compensated liver disease at baseline (Asrani et al., 2014).

### Diffusion weighted imaging

Diffusion weighted imaging (DWI) is a magnetic resonance technique that quantifies the diffusion of water molecules in tissues, and this is quantified as the apparent diffusion coefficient (ADC). The rationale for using this technique to assess liver fibrosis is that the deposition of collagen fibers in the liver would inhibit water diffusion, therefore leading to a decrease in the ADC. There is now considerable experience with this imaging technique, and a meta-analysis of 10 studies reporting the performance of DWI compared to histology, reported pooled AUROCs of 0.86, 0.83, and 0.86 for the diagnosis of any fibrosis (F1-4), significant fibrosis (F2-4) and bridging fibrosis (F3-4), respectively (Wang et al., 2012). This technique has not seen widespread application as it has been demonstrated that confounding factors like steatosis and perfusion also affect the ADC (Luciani et al., 2008; Leitao et al., 2013). Furthermore, when compared with other MR biomarkers of liver fibrosis, DWI was inferior to MRE (Wang et al., 2012) and T_1_ mapping (Cassinotto et al., 2015), and only equivalent to transient elastography and serum based biomarkers (Lewin et al., 2007).

#### T_1_ relaxometry

T_1_ relaxation time is a physical property of atoms that varies according to their electrochemical environment. The T_1_ relaxation time of hydrogen atoms in water molecules is longer that the T_1_ relaxation time of hydrogen atoms in long hydrocarbon chains like fatty acids. Therefore measuring T_1_ can provide information about tissue composition. The observation that T_1_ differed between healthy and diseased livers was made in very early studies of the clinical applicability of MR in visceral organs (Smith et al., 1981; Doyle et al., 1982). Despite this initial promise and subsequent studies in humans that showed some accuracy in the diagnosis of cirrhosis, T_1_ imaging was largely developed for the anatomical assessment of the liver and particularly for the evaluation of liver tumors.

The confounding effects of iron (The Clinical NMR Group, 1987; Hoad et al., 2015) and inflammation / oedema (Chamuleau et al., 1988) on liver T_1_ measures have limited the application of this technique until recently. The current impetus to develop non-invasive assessments of liver disease, combined with enhanced MRI methodologies, have resulted in improvements in the performance of liver T_1_ as a biomarker of fibrosis, with some exciting results. Examples of studies examining the role of T_1_ relaxometry for the assessment of liver fibrosis are outlined in Table 4.

**Table 4:** **T_1_ relaxometry for the assessment of liver disease**.

**Study**	**Study design**	**Patients**	**Main findings**
Smith et al., 1981	HV vs. patients with diffuse liver disease	HV (*n* = 15)	Longer T_1_ in cirrhosis and CAH
		Cirrhosis (*n* = 5)	
		CAH^*^ (*n* = 1)	
Doyle et al., 1982	HV vs. patients with diffuse liver disease	HV (*n* = 12)	Longer T_1_ in cirrhosis
		Cirrhosis (*n* = 10)	
The Clinical NMR Group, 1987	T_1_ vs. histology	Patients with suspected parenchymal disease (*n* = 55)	Tendency toward longer T_1_ in cirrhosis/hepatitis
Thomsen et al., 1990	HV vs. disease controls vs. cirrhosis	HV (*n* = 7)	Longer T_1_ in cirrhosis
		Disease controls (*n* = 17)	No association with histology
	T_1_ vs. histology in a subset		
		Cirrhosis (*n* = 15)	
		Histology subset(*n* = 10)	
Keevil et al., 1994	HV vs. patients with diffuse liver disease	HV (*n* = 42)	Longer T_1_ in patients with cirrhosis and CAH
		Liver disease (*n* = 44)	
Heye et al., 2012	HV vs. cirrhosis	HV (*n* = 31)	T_1_ longer in cirrhosis
		Cirrhosis (*n* = 61)	
Kim et al., 2012	Non cirrhotic patients vs. CHB cirrhosis	Non cirrhotic patients (*n* = 92)	T_1_ shorter in cirrhosis
		HBV cirrhosis (*n* = 87)	
Cassinotto et al., 2015	HV vs. cirrhosis	HV (*n* = 40)	T_1_ longer in cirrhosis
		Cirrhosis (*n* = 89)	
Hoad et al., 2015	T_1_ vs. histology	Training cohort (*n* = 64)	AUROC for cirrhosis 0.92
		Validation cohort (*n* = 46)	
			AUROC for advanced fibrosis 0.81

*HV, healthy volunteers; CAH, chronic active hepatitis; HBV, hepatitis B virus; AUROC, area under the receiver operating characteristic curve*.

* *Chronic active hepatitis is a historical term used to describe a hepatitis of unknown etiology, now believed to be chronic hepatitis C and is no longer used in clinical practice*.

#### Combination of T_1_ relaxation time with other non-invasive biomarkers

In the study by Hoad et al. (2015) liver T_1_ is proposed as a biomarker of liver inflammation, which together with liver T_2_^*^ for liver iron quantification and the ELF panel for fibrosis (William et al., 2004), are combined in a decision tree to determine who should be referred for liver biopsy. This approach resulted in a sensitivity of 90%, specificity of 71%, positive predictive value of 72% and negative predictive value of 89% in identifying those with significant fibrosis or inflammation.

### Dynamic contrast enhanced MRI

Dynamic contrast enhanced (DCE) MRI requires the intravenous injection of hepatocyte specific gadolinium contrast agents like gadoxetic acid (Gd-EOB-DTPA; Primovist©; Bayer, Berlin, Germany). This technique gives an estimate of liver function, and has been shown to differentiate between healthy controls and patients with cirrhosis, and between the cirrhosis severity assessed by Child-Pugh stage or MELD score (Haimerl et al., 2013, 2014; Nilsson et al., 2013; Verloh et al., 2014; Zhao et al., 2015). Retrospective studies have also shown good accuracy for the diagnosis of NASH (AUROC 0.85; Bastati et al., 2014), and for the assessment of fibrosis (Feier et al., 2013; Ding et al., 2015). Furthermore, in a study by Feier et al. fibrosis was the only independent histological predictor of the relative T_1_ change on DCE MRI (Feier et al., 2013).

### Multi-parametric MR protocols

Combinations of several techniques for liver fibrosis assessment have also been examined. In a retrospective study of patients who had undergone liver MR, the combination of parameters derived from DWI, DCE MRI and susceptibility weighed imaging resulted in improved diagnostic accuracy for the classification of fibrosis (AUROC for F0 vs. F1-4: 0.95; F0-1 vs. F2-4: 0.95; F0-2 vs. F3-4: 0.90; F0-3 vs. F4: 0.93; Feier et al., 2016). Incorporation of MR texture analysis techniques with other MR modalities has also produced some promising early results (House et al., 2015; Wu et al., 2015).

#### Iron corrected T_1_

Liver iron is one of the major confounders in the use of liver T_1_ as a biomarker of liver fibrosis, as discussed above. One innovation that has led to improved diagnostic accuracy for liver T_1_ was the development of the liver “iron corrected T_1_” (cT_1_), which removes the confounding effect of iron from the T_1_ measurements making it much more widely applicable (Tunnicliffe et al., 2014). In a prospectively recruited cohort of patients undergoing liver biopsy for the assessment of fibrosis, cT_1_ showed excellent diagnostic accuracy against the Ishak staging system of fibrosis with an AUROC of 0.94 for the diagnosis of patients with any degree of fibrosis (F0 vs. *F*≥1; Banerjee et al., 2014). Furthermore, liver cT1 was used to derive the liver inflammation and fibrosis (LIF) score, a standardized continuous (0–4) score recently shown to predict liver related clinical outcomes, and is thus of potential use in predicting prognosis in patients with chronic liver disease (Pavlides et al., 2016).

### Molecular MR imaging

Molecular MR imaging represents a unique implementation of MR technology to visualize biological processes at the cellular and molecular level. Studies examining these technologies are still restricted to animal models and it remains to be seen whether they are safe and clinically applicable in humans.

Type I collagen is a major constituent of liver fibrosis. As it deposited in the extracellular space, it is an ideal target for molecular MR imaging. A molecular probe (EP-3533) to type I collagen has been developed, and is the most extensively studied probe in molecular MR imaging of liver fibrosis. When injected, this probe leads to shortening of the T_1_ relaxation time. In a feasibility study (Polasek et al., 2012), animals with fibrosis were found to have greater signal intensity compared to controls (0.55 vs. 0.39; *p* < 0.05) when imaged after EP-3533 injection. Furthermore, EP-3533 content measured in the sacrificed animals had a strong correlation with Ishak staging (*r* = 0.79 to 0.84 depending on the animal model). A subsequent study demonstrated that EP-3533 was superior to other MR biomarkers of fibrosis (T_1_ relaxation time, T_1_ρ, DWI and magnetisation transfer techniques; Fuchs et al., 2013). In their latest study, the group used a clinical scanner and showed that the technique can be useful in monitoring therapeutic effects using the bile duct ligation animal model of fibrosis (Farrar et al., 2015). Control animals, or animals that responded to rapamycin developed less fibrosis and had lower enhancement after EP-3533 compared to animals that did not receive or did not respond to treatment.

## Conclusion

In summary, this review serves to provide a reference for the application of both serum and imaging based non-invasive markers of liver fibrosis. The use of these important clinical adjuncts has been discussed in detail, although given the plethora of non-invasive markers reported in the literature, the authors have specifically focused on the most well-known and available tools. While serum based algorithms and established elastography methods are being validated in large clinical cohorts of different liver diseases and demonstrate important predictive power in detecting liver related outcomes, imaging methodologies evolve apace, with promising novel approaches giving simultaneous diagnostic, staging as well as prognostic information entering the non-invasive arena.

## Author contributions

JR devised, wrote and revised the manuscript, provided funding for open access. JC, MP, and AM wrote sections of the manuscript and critically reviewed the entire paper.

## Funding

Oxford Health Services Research Committee (OHSRC) grant (Ref: EC/1163).

### Conflict of interest statement

MP is a shareholder of Perspectum Diagnostics, an Oxford University spin-out company. The other authors declare that the research was conducted in the absence of any commercial or financial relationships that could be construed as a potential conflict of interest.
